# High-speed photon-counting laser ranging for broad range of distances

**DOI:** 10.1038/s41598-018-22675-1

**Published:** 2018-03-08

**Authors:** Bingcheng Du, Chengkai Pang, Di Wu, Zhaohui Li, Huan Peng, Yuliang Tao, E. Wu, Guang Wu

**Affiliations:** 1 State Key Laboratory of Precision Spectroscopy, East China Normal University, Shanghai, 200062 China; 2 Beijing Institute of Space Mechanics & Electricity, Beijing, 100091 China; 3 Collaborative Innovation Centre of Extreme Optics, Shanxi University, Taiyuan, 030006 China

## Abstract

We demonstrate a high-speed photon-counting laser ranging system with laser pulses of multiple repetition rates to extend the unambiguous range. In the experiment, the laser pulses of three different repetition rates around 10 MHz were employed to enlarge the maximum unambiguous range from 15 m to 165 km. Moreover, the range of distances was increased as well, enabling the measurement on different targets of large separation distance with high depth resolution. An outdoor photon-counting laser ranging up to 21 km was realized with high repetition rate, which is beneficial for the airborne and satellite-based topographic mapping.

## Introduction

Nowadays, the airborne LiDAR (Light Detection and Ranging) has promoted the applications of remote sensing in the fields of three-dimensional (3D) city modeling, forestry, electricity, sea fog and so on^1–5^. Meanwhile, single-photon detections have been used in the time-of-flight (TOF) laser ranging and imaging systems based on the time-correlated single-photon counting (TCSPC) technique^6–9^, which is one of the promising candidates for a practical airborne LiDAR. Such systems transmit low energy laser pulses and detect the return photons from the non-cooperative targets^10–12^, establishing the time correlation between the trigger signals and the receiving photons to obtain the accurate distance information of the targets at kilometre scale, which have been implemented in a great deal of different fields such as airborne platforms^13^, satellite and aircraft observations^14^, topographic mapping^15^, and so forth. Especially, the TCSPC-based laser ranging system is very suitable for the airborne LiDAR which is usually about 10 km above the ground, because the portable and compact semiconductor laser sources can be used in the system to produce the low-energy laser pulses and the rapid acquisition of the TOF data can be achieved with the high laser pulse repetition rate. Note that the range ambiguity is one of the key parameters in the TCSPC-based laser ranging system for the unknown targets and rapidly-moving targets because the TOF of return photons is indeterminate when it is beyond one period of the laser pulse. Then, the maximum unambiguous distance *d*_max_ is limited by

1 $${d}_{max}=\frac{c}{2f}=\frac{c\cdot T}{2},$$

where *c* is the speed of light in the transmission media, *f* is the repetition rate of the pulsed laser, and *T* is the period time. Although low repetition rate would result in long maximum unambiguous distance, LiDAR systems with high repetition rate are favored for the measurement on the fast-moving targets due to the short acquisition time. Therefore, up to now, various approaches have been implemented to increase the unambiguous distance in the high-speed TCSPC-based ranging systems, such as the pseudo-random pattern matching scheme^16^ where the target’s distance information could be determined by the correlation between the temporal patterns of the transmitted and the return pulses while the absolute range could be extended by adding the length of the periodic patterns. Meanwhile, multi-repetition-rate scheme has also been demonstrated to increase the maximum unambiguous distance in a high-speed photon-counting laser ranging system^17,18^. In our previous work, we developed a ranging system at 1550 nm using two different laser repetition rates around 10 MHz to extend the maximum unambiguous distance from 15 m to ~1.5 km^17^.

In this paper, we employed the laser pulses of three different repetition rates as the light source in the photon-counting laser ranging system to improve the ranging capability of the system. The maximum unambiguous distance was enlarged to be about 165 km. We demonstrated an 11-km ranging in the urban area and a 21-km imaging experiment in field use. Owing to the enlarged unambiguous distance, a broad range of distances was achieved in the measurement with high depth resolution within one acquisition window, which could fulfil the requirement of the rapid LiDAR system for the long-distance range.

## Results

### Maximum unambiguous distance

In the multi-repetition-rate TCSPC-based laser ranging scheme^17,18^, assuming that each period of the laser repetition rate are *T*_1_, *T*_2_… *T*_n_, respectively, the maximum unambiguous distance *d*_max_ can be derived by

2 $${d}_{max}=\frac{c\cdot [{T}_{1},{T}_{2}\mathrm{...}\,{T}_{n}]}{2},$$

where [*T*_1_, *T*_2_… *T*_n_] is the least common multiple of *T*_1_, *T*_2_… *T*_n_, and each periods should be integers in mathematics. In our experiment, the coprime periods of 100 ns, 103 ns and 107 ns were chosen so that the repetition rate of the laser pulses was around 10 MHz while the maximum unambiguous distance was up to 165.315 km according to equation (2). The actual distance *L* (≤*d*_max_) of the target is determined by

3 $$L=\frac{c\cdot ({q}_{1}{T}_{1}+{t}_{1})}{2}=\frac{c\cdot ({q}_{2}{T}_{2}+{t}_{2})}{2}=\cdot \cdot \cdot =\frac{c\cdot ({q}_{n}{T}_{n}+{t}_{n})}{2},$$

where *q*_1_, *q*_2_… *q*_n_ are the single-valued period numbers of the different periods, and *t*_1_, *t*_2_… *t*_n_ are the photon correlation time recorded by the TCSPC system. *L* can be finally obtained by substituting the values of *T*_1_, *T*_2_… *T*_n_ and *t*_1_, *t*_2_… *t*_n_ into equation (3).

### An 11-km ranging experiment in the urban area

**Figure 1:**
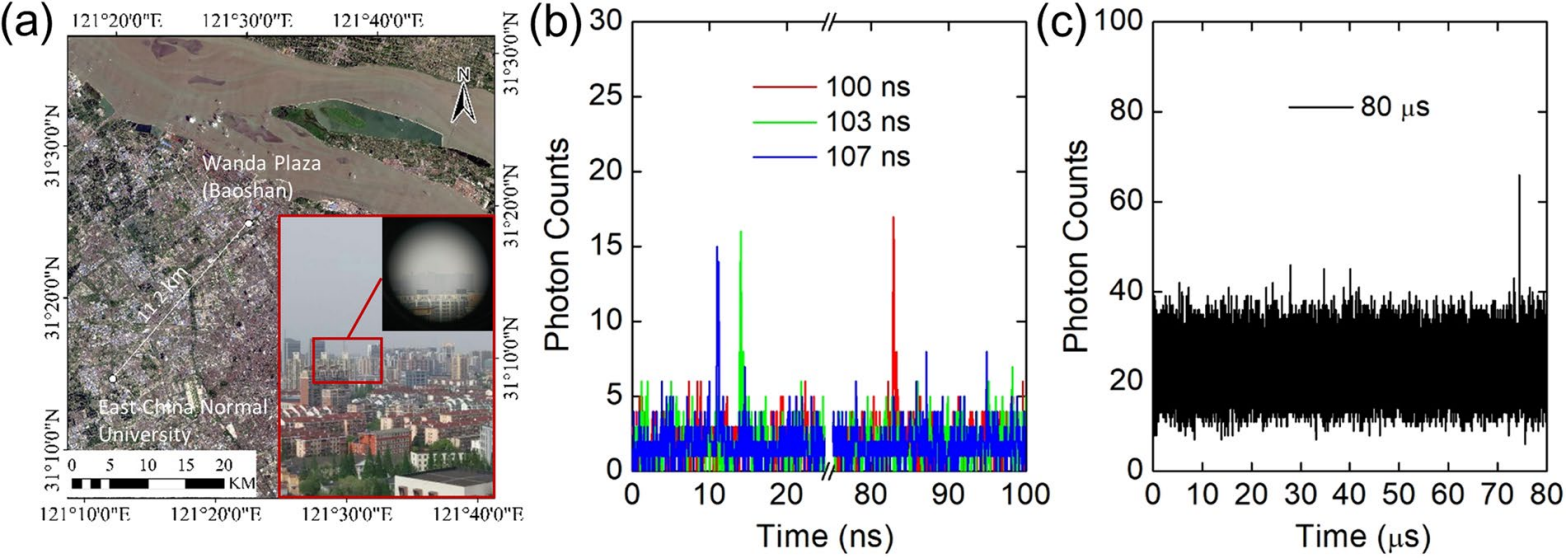
Results of the 11-km ranging experiment. (**a**) Distance between the transmitter and the target in the map. ArcGIS 10.4 software (http://www.esri.com) was used to generate the figure. The data source was extracted from Landsat-7 images by courtesy of the U.S. Geological Survey (https://www.usgs.gov, NASA Landsat Program, 2017, Landsat OLI_TIRS scenes LC08_L1TP_118038_20170824_20170912_01_T1, L1TP, USGS, Sioux Falls, 09/12/2017). Inset: real view of the target taken by a camera with a telescope. (**b**) Histogram of return photons from the target with the acquisition time of 0.1 s with pulsed laser source of three different repetition rates around 10 MHz. (**c**) Histogram of return photons from the target with the acquisition time of 30 s with laser source of single repetition rate of 12.5 kHz.

Figure 1(a) shows the distance between the photon-counting laser ranging system and the target in the map, which is about 11.2 km. In the telescope photo as the inset of Fig. 1(a), the target building is hardly observable due to the long distance. The experiment was carried out at night with the atmospheric visibility of 10.2 km. The multi-repetition-rate TCSPC-based laser ranging system is described in Method. We operated the system at three different repetition rates independently for acquisition. The periods of the laser pulses were 100 ns, 103 ns and 107 ns, respectively. The resolution of the TCSPC was set at 64 ps. The overall time jitter of the system output was about 224 ps. Figure 1(b) shows the histograms of the return photons recorded by the TCSPC-based system when the acquisition time was set to be 0.1 s. The signal photon-counting peaks locate at 10.98 ns, 13.92 ns and 82.81 ns for the laser periods of 100 ns, 103 ns and 107 ns, respectively. According to equation (3), the target distance is calculated to be 11173.44 m. Meanwhile, In order to verify the reliability of the laser ranging system, a comparison experiment on the same target was conducted with the laser pulse repetition rate at a single value of 12.5 kHz (the period of 80 μs) as shown in Fig. 1(c). The timing resolution of TCSPC had to be increased to 2 ns for the limited acquisition window and the acquisition time was set to be 30 s to accumulate enough signal photon counts. According to the arrival time of the return photons, the target distance was 11172.4 m. The measured distance was consistent with the previous results, proving the accuracy of the multi-repetition-rate scheme. Comparing the two methods, the acquisition time can be much shortened to accumulate enough useful signal photons to obtain the TOF analysis in the multi-repetition-rate scheme since the repetition rate is much higher.

### A 21-km imaging experiment in field use

**Figure 2:**
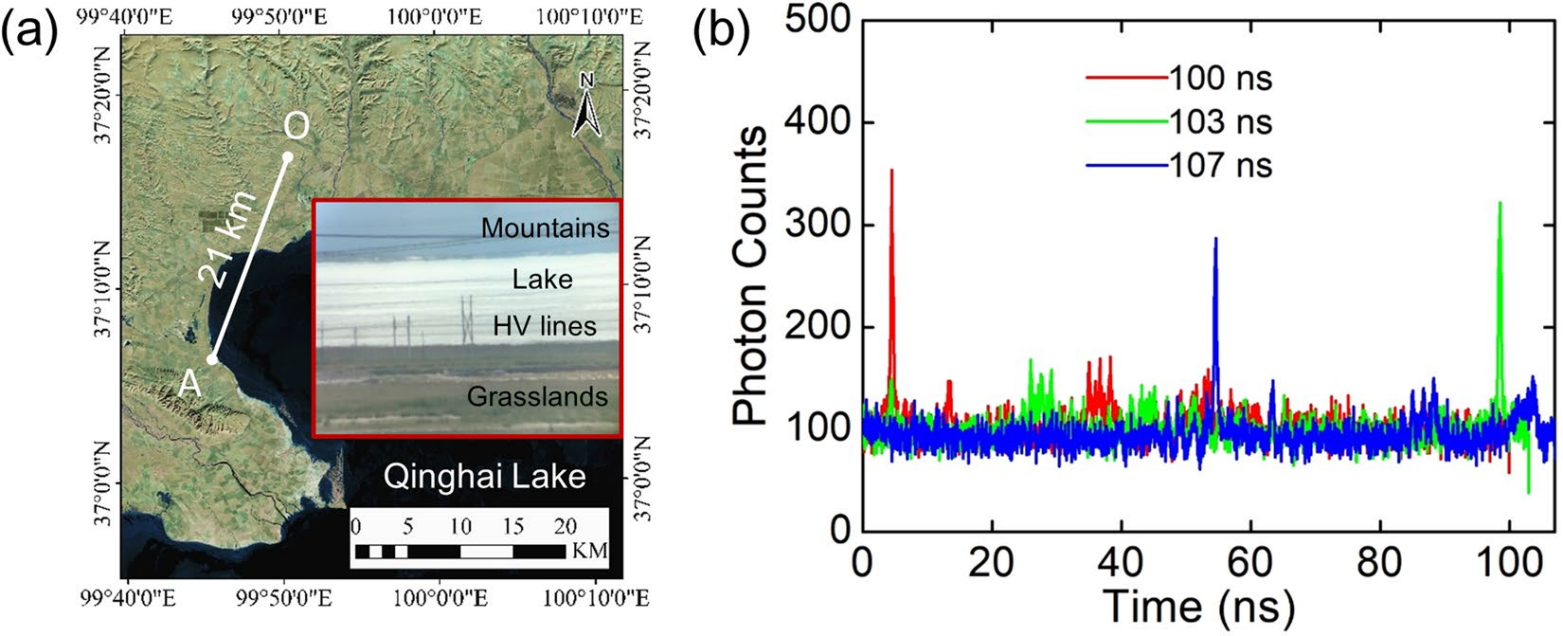
Results of the 21-km ranging experiment. (**a**) Distance of the observation location and Spot A across Qinghai Lake in the map. ArcGIS 10.4 software (http://www.arcgis.com) was used to generate the figure. The data source was extracted from Landsat-7 images by courtesy of the U.S. Geological Survey (https://www.usgs.gov, NASA Landsat Program, 2017, Landsat OLI_TIRS scenes LC08_L1TP_133034_20170105_20170312_01_T1, L1TP, USGS, Sioux Falls, 03/12/2017). Insert: photo of a real scene of Spot A in the observation point. HV lines: high-voltage lines. (**b**) Histogram of return photons from the target 21 km away with the acquisition time of 10 s by three laser repetition rates.

We carried out a long-distance photon-counting laser ranging with the multi-repetition-rate system in field use. The system was transported from the laboratory to the demonstration location by car via 2000-km highway and installed on the top floor of an inn close to Qinghai Lake as shown by the circle in Fig. 2(a). The whole system showed high robustness in the long distance transportation. In the demonstration, the single pulse energy at the laser output was increased to 4.0 × 10^−8^ J. The divergence angle of the laser beam was reduced to about 0.08 mrad by extending the beam size to be 40 mm at the output of the telescope. We set the same parameters for the laser periods and the resolution of the TCSPC but the acquisition time was increase to be 10 s. Figure 2(b) shows a TOF measurement on the target at Spot A. The signal photon-counting peaks locate at 4.51 ns, 98.53 ns and 54.62 ns for the laser periods of 100 ns, 103 ns and 107 ns, respectively. According to equation (3), the target distance is calculated to be 21675.60 m, which shows the precise measurement of the multi-repetition-rate system in field use.

**Figure 3:**
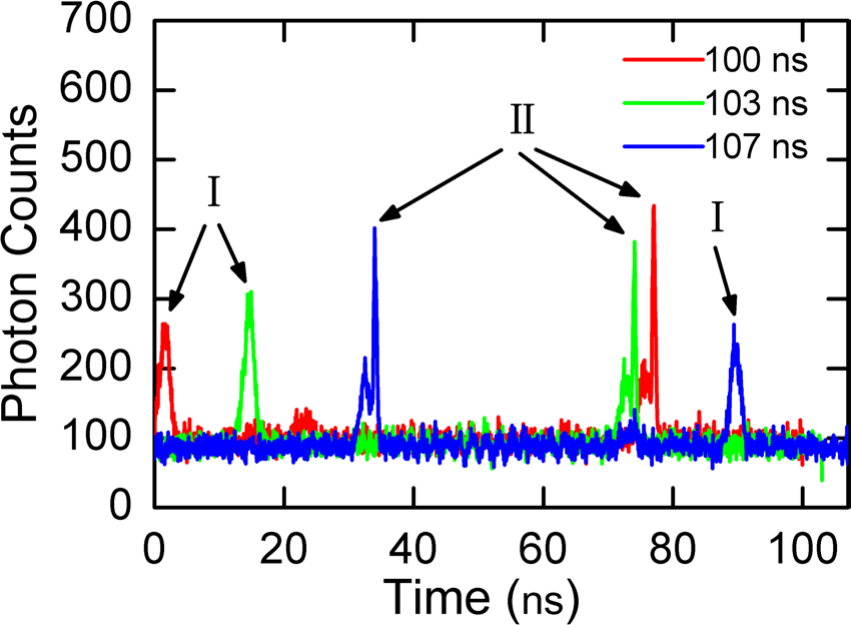
Histogram of return photon counts from two targets of different distances within one acquisition window. (Target I: ~5.1 km, Target II: ~21 km).

In field use, there was a great variety of targets at different distances, including the nearby high-voltage (HV) lines, grasslands and mountains across the lake at Spot A as shown in the inset of Fig. 2(a). As the unambiguous range was increased, the measurement range of the system was enlarged as well, offering the opportunity to observe two targets of different distances in one acquisition by using the multi-repetition-rate ranging system. Figure 3 shows the TOF histograms from two targets at different distances within one acquisition window. In each curve, two signal peaks could be observed. The timing interval of the two peaks was independent. The lower peaks in each curve represent the same Target I, which is determined to be 5128.35 m from the observation location. And the higher peaks in each curve determine the distance of Target II to be 21677.85 m. According to Fig. 2(a), we find that Target I matches with the nearby HV lines, and Target II matches with the mountain across the lake. In the common LiDAR system, the measurement range is limited by the repetition rate of the laser pulse. Since the acquisition window and the resolution of the TCSPC are contrary parameters, we cannot obtain a high resolution TOF measurement for very long distance with a large acquisition window for a given TCSPC system without. But in the multi-repetition-rate scheme, a large measurement range can be easily obtained in the long distance TOF measurement with high depth resolution. Owing to the enlarged unambiguous range and the broad range of distances, two targets separated by 16 km could be measured in one acquisition with high resolution.

**Figure 4:**
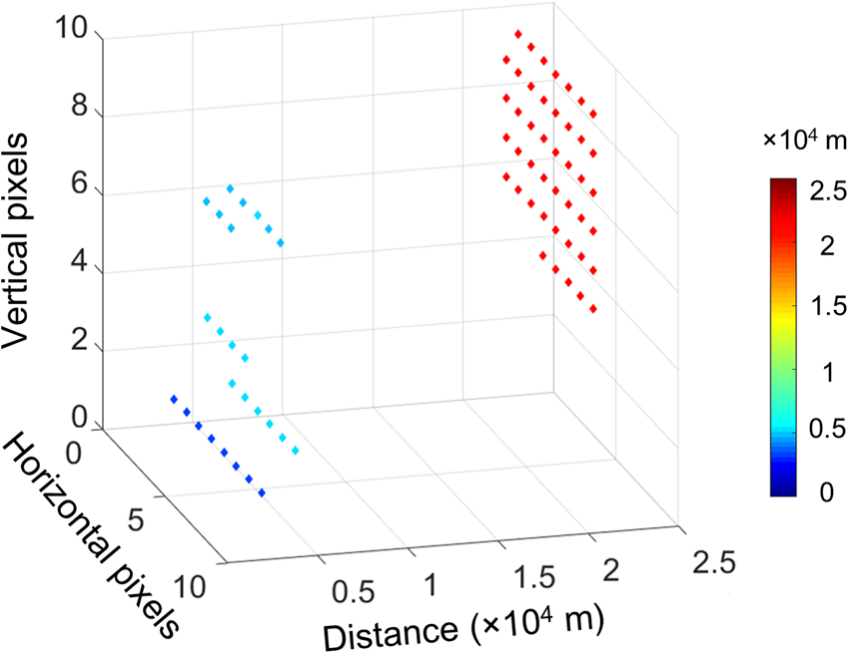
Scanning 3D image with 8 × 10 pixels of Spot A. Each dot in the image represents the target distance and the corresponding horizontal and vertical position. The color bar indicates the distance of the targets.

In order to verify the possibility of implementing the system in a 3D imaging for long distance, we demonstrated a scanning imaging experiment with 8 × 10 pixels toward Spot A. We installed the system on a 2D rotation stage. The scanning resolution of the rotational plate was set at 0.01°/pixel both in the horizontal and the vertical directions. All the acquisition parameters remained the same as in the previous experiment. As shown in Fig. 4, targets of different distances could roughly be recognized and matched with the scene in the inset of Fig. 2(a). The blue dots around 3–5 km correspond to the HV lines. The red dots around 21 km represent the mountains. There is a blank from 5 to 21 km, indicating the existence of the lake since most of the laser beams were transmitted on the lake without any returning photon signals. Owing to the broad range of distances, the nearby HV lines and the mountains far away could be imaged within one acquisition, showing in Row 6 and 7 of the image, indicating that the multi-repetition-rate LiDAR system is suitable for long distance 3D imaging for complex targets.

## Conclusions

We demonstrated a high-speed long-distance photon-counting laser ranging system in field use. By implementing three different repetition rates around 10 MHz to modulate the laser source, the unambiguous distance could reach 165 km. Moreover, a broad range of the measurement was achieved within one detection window, enabling to image targets with large distance difference rapidly in one acquisition. This system can be applied to the airborne or satellite-based laser ranging system and the full-waveform LiDAR with long-distance range and high speed.

## Methods

**Figure 5:**
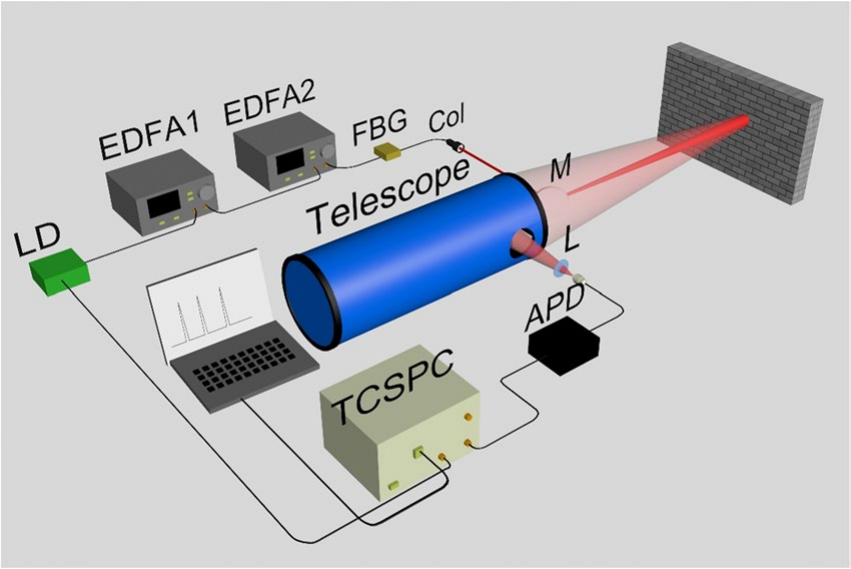
Experimental setup of the laser ranging system with multi-repetition-rate laser pulses. LD: laser diode at 1550 nm; EDFA1, 2: erbium-doped fibre amplifiers; FBG: fibre Bragg grating; Col: collimator; M: high-reflection mirror; L: convex lens; APD: InGaAs/InP APD; TCSPC: time-correlated single-photon counting system (PicoHarp300, PicoQuant GmbH, Germany).

The schematic diagram of the experimental setup is shown in Fig. 5. The light source was a fibre-coupled pulsed laser diode at 1550 nm. The laser diode was modulated by a short-pulse circuit, and the circuit was synchronized to a function generator (AFG3252, Tektronix, INC) which also triggered the “Start” of the TCSPC. The laser power was two-stage amplified by the EDFAs to achieve the overall amplification factor of 38.2 dB. The amplified spontaneous emission was suppressed by a fibre Bragg grating with the centre wavelength at 1549.9 nm and the linewidth of 1.0 nm. After that, the amplified laser beam was collimated into the free space by a collimator (RC08APC-P01, Thorlabs, INC). The single pulse energy was about 2.0 nJ at the output of the collimator, and the divergence angle of the laser beam was about 0.35 mrad. By using a high-reflection mirror, the transmitted laser beam was coaxial with the receiving Newtonian telescope with the diameter of 130 mm, which permitted the system to collect adequate scattered return photons. The return photons were fibre-coupled into the fibre-pigtailed InGaAs/InP avalanche photodiode (APD) with the fibre core of 62.5 µm in diameter. The single-photon detector was operated in quasi-continuous Geiger mode with sinusoidal gating waves at 1 GHz^17^. The InGaAs/InP APD was Peltier cooled to 240 K. The detection efficiency was 3.58% at 1550 nm, the dark count rate was 2.0 × 10^−5^ counts/gate. The output of the single-photon detector was connected to the “Stop” of the TCSPC while the synchronous trigger signal from the function generator was connected to the “Start” of the TCSPC. The temporal resolution of the TCSPC was selected to be 64 ps. The time interval between the “Start” and the “Stop” signals presented the flight time of photons in one period. The overall time resolution of the system output was about 224 ps, including the influence from the laser pulse duration, the timing jitter of the single-photon detector and the temporal resolution of the TCSPC. The depth resolution was determined to be ~3.4 cm according to the overall time resolution of the system output of ~224 ps.

### Data Availability

The datasets generated during the current study are available from the corresponding author on reasonable request.
